# Role of oxidative stress in the relationship between periodontitis and systemic diseases

**DOI:** 10.3389/fphys.2023.1210449

**Published:** 2023-07-12

**Authors:** Jiaxin Shang, Haifeng Liu, Youli Zheng, Zheng Zhang

**Affiliations:** ^1^ Tianjin Stomatological Hospital, School of Medicine, Nankai University, Tianjin, China; ^2^ Tianjin Key Laboratory of Oral and Maxillofacial Function Reconstruction, Tianjin, China; ^3^ The School and Hospital of Stomatology, Tianjin Medical University, Tianjin, China

**Keywords:** periodontitis, oxidative stress, systemic diseases, reactive oxygen species, inflammation

## Abstract

Periodontitis is a common inflammatory disease. It is characterized by destruction of the supporting structures of the teeth and could lead to tooth loss and systemic inflammation. Bacteria in inflamed gingival tissue and virulence factors are capable of entering the bloodstream to induce systemic inflammatory response, thus influencing the pathological process of many diseases, such as cardiovascular diseases, diabetes, chronic kidney disease, as well as liver injury. An increasing body of evidence show the complex interplay between oxidative stress and inflammation in disease pathogenesis. When periodontitis occurs, increased reactive oxygen species accumulation leads to oxidative stress. Oxidative stress contributes to major cellular components damage, including DNA, proteins, and lipids. In this article, the focus will be on oxidative stress in periodontal disease, the relationship between periodontitis and systemic inflammation, and the impact of periodontal therapy on oxidative stress parameters.

## 1 Introduction

Periodontitis is a dysbiotic disease characterized by an imbalance of the microbial community within the periodontal tissues, leading to chronic inflammation and destruction of the tooth’s supporting structures (Eriksson et al., 2019; Inanc et al., 2021; Giannini et al., 2022). As a common inflammatory disease, periodontitis affects 10%–15% of adults and can eventually lead to tooth loss (Rajbhandari and Shrestha, 2018). It may have significant implications on an individual’s oral and systemic health. The current understanding of periodontitis has shifted from clinical parameters to the pathogenesis of the disease, and the involvement of microbial composition, immune response, and genetic susceptibility.

The etiopathogenesis of periodontitis is complex, involving both host and microbial factors (Maulani et al., 2021). Dysbiosis, or microbial imbalance, is thought to be the primary driver of periodontitis and is characterized by an overabundance of pathogenic bacteria and a reduction in symbiotic bacteria (Na et al., 2020). The virulence factors of periodontal bacteria include lipopolysaccharides (LPS), proteases, and other enzymes that disrupt the host immune response and promote tissue destruction (Wang H. Y. et al., 2017; Zhou et al., 2023). LPS is highly immunogenic and has the ability to induce the production of pro-inflammatory cytokines. Proteases are involved in the destruction of extracellular matrix and host immune proteins (Blasco-Baque et al., 2017). Other bacterial virulence factors, including fimbriae, capsules, and toxins, as well as host variables such as genetic susceptibility and systemic diseases like diabetes, also contribute to the development of periodontitis (Xu et al., 2020).

Periodontitis is usually associated with activation of polymorphonuclear leukocytes, which in turn may generate reactive oxygen species (ROS) during inflammatory conditions (Hatipoğlu et al., 2015). Oxidative stress is a complex biological process characterized by the excessive production of ROS, which act as destroyers to the redox balance in body and induce oxidative damage (Rotariu et al., 2022). All the metabolisms are impaired in oxidative stress and even nucleic acid balance is influenced. ROS causes oxidative damage to the tissues via multiple mechanisms, including DNA damage, protein oxidation and lipid peroxidation (LPO) damage (Heinkele et al., 2021). Periodontitis is associated epidemiologically with several chronic diseases, such as cardiovascular disease, type 2 diabetes mellitus (T2DM) and nonalcoholic fatty liver disease. Oxidative stress plays an important role in the impact of periodontitis on systemic disease (Ye et al., 2012). The following is an overview of research on the relationship between oxidative stress, periodontitis, and systemic disease.

## 2 Oxidative stress

Oxidative stress is an imbalance between the production of ROS and the antioxidant capacity of cells, which damages biological systems due to increased ROS production and dysfunction of the antioxidant system (Ibrahim et al., 2020; Kim et al., 2021). Under physiological conditions intracellular ROS are normal components of signal transduction cascades. And the levels of ROS are maintained by a complex antioxidants systems participating in the *in-vivo* redox homeostasis (Obeng-Gyasi, 2018). However, inflammatory responses can also be stimulated by ROS through protein kinases, transcription factors, and genomic expression of inflammatory factors genomic expression (Besednova et al., 2022). Increased ROS accumulation leads to oxidative stress, which contributes to major cellular components damage, including DNA, proteins, and lipids (Perera et al., 2020; Jia et al., 2021).

Oxidative DNA lesions can be formed through two distinct pathways, including: 1) direct oxidation of a base (purine/pyrimidine) in DNA; 2) misincorporation of oxidized deoxy nucleoside triphosphates into DNA by DNA polymerase. All four bases of the DNA can undergo direct oxidation, forming various oxidized purines. Among the various forms of oxidative DNA damage, 8-oxoDG and 8-hydroxy-2′deoxyguanosine (8-OHdG) are the most studied and recognized markers of oxidative DNA alterations (Caliri et al., 2021).

Oxidative damage to proteins is divided in two categories, including the reversible and irreversible protein modifications (Caliri et al., 2021; Fu et al., 2022). Protein carbonylation is an irreversible protein modification, resulting from oxidative damage, that often leads to loss of protein function (Matsuo et al., 2021). The specificity of multiple amino acids to undergo carbonylation has made this modification a widely used biomarker for assessing oxidative damage to proteins (Caliri et al., 2021). Reversible oxidation of proteins with adjacent cysteine residues, possibly including protein kinases and phosphatases, can regulate protein function and redox signaling pathways in various stress responses (Caliri et al., 2021; Sologova et al., 2022).

ROS can induce degradation of polyunsaturated fatty acids resulting in the formation of a variety of products (Caliri et al., 2021; Emanuelli et al., 2022). Lipid peroxidation can directly damage phospholipids to form oxidized phospholipids, which induces cell death through apoptosis, necrosis, pyroptosis, or ferroptosis, and involved in a variety of inflammatory responses (Zhu et al., 2022). Biomembranes are prone to undergo lipid peroxidation, and it is possibly via two pathways: non-enzymatic and enzymatic (Su et al., 2019; Ruan et al., 2021). The non-enzymatic pathway is an iron-dependent lipid peroxidation (Chen et al., 2022). The enzymatic pathway involves a highly organized oxygenation center, wherein oxidation occurs on only one class of phospholipids (Su et al., 2019).

## 3 Oxidative stress and periodontitis

After host defense responses are triggered by periodontal pathogenic bacteria in biofilm, neutrophils become the most common inflammatory cells that accumulate in periodontal tissue and gingival sulcus (Chu et al., 2021). Neutrophils are believed to be the predominant sources of ROS in periodontitis (Wang Y. et al., 2017). During phagocytosis of periodontal pathobionts, neutrophils can release excess ROS via the NADPH oxidase pathway (Sui et al., 2020). However, ROS has a very short half-life, and it is not easy to be detected. Therefore, ROS-related degradants and enzymatic and non-enzymatic antioxidant activity are ideal candidates for assessing the impact of oxidative stress-related events on the pathological process of periodontitis (Monmeesil et al., 2019). Changes in local concentrations of oxidative stress biomarkers are closely associated with the progression of periodontitis. It suggested that the oxidative stress biomarker level can be used for periodontitis diagnosis and therapeutic efficacy evaluation (Wang Y. et al., 2017).

Various explanations have been offered for the relationship between the concentration of local markers of oxidative stress and the progression of periodontitis. For example, higher levels of malondialdehyde (MDA), hydrogen peroxide, and oxidative DNA damage have been reported in patients with periodontitis (Wu et al., 2016). Several studies have shown that decreased activity of enzymatic antioxidants such as superoxide dismutase (SOD) and catalase (CAT) is associated with periodontitis (Almerich-Silla et al., 2015). The study also showed significant differences in the levels of oxidative stress biomarkers (total antioxidant capacity, MDA, glutathione peroxidase, nitric oxide, total oxidative status, and 8-hydroxydeoxyguanosine) at the site between patients with periodontitis and healthy controls (Almerich-Silla et al., 2015).

The pathogenesis of periodontal tissue destruction is believed to involve oxidative stress (Zhang et al., 2022). Almerich-Silla et al. (2015) raised that oxidative stress levels in periodontium were significantly higher in the periodontal disease groups than in the gingivitis groups and healthy groups, the oxidative stress showed a linear trend associated with periodontal worsening as well as bleeding on probing (BOP). Patients with periodontitis had elevated levels of biomarkers of ROS-induced tissue damage and elevated levels of antioxidant enzymes corresponding to oxidative stress in inflamed periodontal tissue and gingival fluid (De Angelis et al., 2022).

Recent studies have focused on the involvement of ROS in the pathogenesis of periodontitis, focusing on apoptosis of human periodontal ligament stem cells (hPDLSCs), migration of periodontal ligament fibroblasts (PDLFs), and alveolar bone loss (Sui et al., 2020). At the right concentration, ROS plays a key role in cell proliferation, migration, apoptosis, and wound healing (Tottoli et al., 2020; Cordani et al., 2021). ROS can increase the expression of dynamin-related protein 1 (Drp1), a key regulator of mitochondrial fission, leading to mitochondrial dysfunction, including abnormal mitochondrial membrane potential and reduced ATP levels, ultimately leading to hPDLSC apoptosis (Sui et al., 2020). In addition, at low concentrations, ROS may stimulate proliferation and differentiation of PDLF in culture. While ROS at high concentrations may have cytotoxic effects on periodontal tissue (Liu et al., 2017).

Periodontitis is triggered by a shift in the oral microbiome towards a community enriched in anaerobic Gram-negative bacteria (Munteanu et al., 2022). These microorganisms are equipped with intrinsic virulence factors, including endotoxin and LPS, which is the main constituent of the Gram-negative bacterial outer membrane. Upon its release, LPS triggers a complex immune response mediated by a variety of host-derived factors that perpetuate the inflammatory processes (Jia et al., 2019; Sidhu et al., 2020). Additionally, LPS may activate immune cells, supplementing the generation of bioactive molecules such as ROS, leading to oxidative stress and further destruction of periodontal tissues (Vo et al., 2020). The contributions of LPS and oxidative stress are crucial in the pathogenesis of periodontitis. LPS can stimulate the generation of proinflammatory cytokines, which provoke and sustain immune cell recruitment and activation as well as tissue damage in periodontal tissues (Han et al., 2022). Concurrently, oxidative stress causes direct harm to cells and tissues, leading to further tissue damage, as well as inflammatory responses that increase the microbial dysbiosis (Liu et al., 2023). Cumulatively, the shift towards an anaerobic Gram-negative bacterial community, along with the presence of LPS and oxidative stress, act synergistically to promote chronic inflammation and to foster the exacerbation of periodontal diseases (Willmann et al., 2018). Liang et al. found that treatment of PDLF with LPS overproduces ROS and induces the binding of thioredoxin (TXNIP) and NOD-like receptor protein 3 (NLRP3) to form NLRP3 inflammasomes (Sui et al., 2020). As intracellular signaling transduction molecules, ROS also promote osteoclast formation, leading to alveolar bone resorption and periodontal tissue damage (Sui et al., 2020).

Experiments have shown that antioxidants can mitigate the irreversible teeth-supporting tissues damage caused by excess ROS. Local vitamin C (an important water-soluble vitamin with antioxidant and immunomodulatory properties) inhibits inflammatory resorption by the alveolar bone and reduces oxidative stress and tissue destruction induced by inflammation (Toraman et al., 2020). Local vitamin C may be a therapeutic agent that can be used in the treatment of periodontitis (Toraman et al., 2020). Proanthocyanin, a potent grape seed antioxidant, has been reported to reduce inflammation and alveolar bone loss due to periodontitis by decreasing HIF-1α and MMP-8 levels and increasing osteoblast activity in diabetic rats periodontitis (Toker et al., 2018). In a study of rats with ligamentous periodontitis, melatonin treatment appeared to suppress the production of inflammatory cytokines and relieve gingival inflammation (Permuy et al., 2017; Li L. et al., 2021). The authors concluded that melatonin could reduce oxidative stress and periodontal inflammation by decreasing the levels of inflammatory cytokines and restoring antioxidant levels in the tissues (Permuy et al., 2017; Li L. et al., 2021). Results of a murine periodontitis model clearly demonstrated that polydopamine nanoparticles could remove ROS and decrease the periodontal inflammation as robust antioxidants (Li Q. et al., 2021).

The above studies show a close relationship between periodontitis and oxidative stress, with periodontitis triggering the mechanism of oxidative damage and oxidative stress influencing the development of periodontitis and further aggravating the damage to periodontal tissues.

## 4 Periodontitis and systemic diseases interrelationships: role of oxidative stress

Periodontitis causes systemic inflammation and oxidative stress, which can lead to a number of diseases (Kurek-Gorecka et al., 2022; Xu et al., 2022; Yeh et al., 2022). Bacteria in inflamed gingival tissue and virulence factors are capable of entering the bloodstream to induce systemic inflammatory response, thus influencing the pathological process of many diseases, such as cardiovascular diseases, diabetes, chronic kidney disease, as well as liver injury.

### 4.1 Periodontitis and cardiovascular diseases

The impact of oxidative stress on cardiovascular disease is a hot topic of research. Oxidative stress may be one of the factors that explain the pathophysiological mechanisms of inflammatory conditions in cardiovascular disease and periodontitis (Liu et al., 2017). Persistent systemic inflammation due to periodontitis can lead to vascular endothelial dysfunction and increase inflammation in existing atherosclerotic lesions, which increases the risk of cardiovascular diseases and related events (Stanescu et al., 2020). Oxidative stress is associated with the development of coronary atherosclerotic complications and various risk factors (Corredor et al., 2022). It is reported that ROS can triggers immune responses through redox-sensitive gene transcription factors, such as nuclear factor-κB (NF-κB), leading to the expression of inflammatory cytokines (Liu et al., 2017). Studies have shown that periodontitis is associated with excessive ROS production in periodontal tissue, gingival crevicular fluid (GCF) or gingival blood (Corredor et al., 2022). The systemic effects of periodontitis are due to the diffusion of ROS produced in periodontal lesions into the blood stream.

Study suggests that periodontitis is a potential risk factor for acute myocardial infarction (AMI) (Turgut Cankaya et al., 2018; Kregielczak et al., 2022). Circulating lipid peroxides related to periodontitis were found in both AMI and control subjects (Diaz et al., 2020; Toczewska et al., 2020). The study also suggests that oxidative stress could be the main pathogenic link between AMI and periodontitis (Diaz et al., 2020). In patients who are affected by cardiovascular disease or periodontitis, the condition of low Coenzyme Q10 (CoQ10) levels has been reported (Ferlazzo et al., 2021). This compound is a cofactor, that is, involved in the production of ATP in the mitochondrial respiratory chain. It takes part in redox reactions and plays a role as an antioxidant by reducing ROS (Ferlazzo et al., 2021). Subjects with periodontitis and coronary heart disease showed a significant increase in asymmetric dimethylarginine levels (Toczewska et al., 2020; Giannini et al., 2022). In periodontitis and coronary heart disease subjects, the author observed an increased concentration of nitrotyrosine, associated with lower levels of CoQ10 in comparison to controls (Ferlazzo et al., 2021).

In an animal experiment, the effects of caffeic acid phenethyl ester on alveolar bone resorption, cytokine levels, and oxidative status were assessed by using a rat model of periodontitis, and suggests that periodontal infection may affect the heart by increasing inflammatory and oxidative responses (Otan Ozden et al., 2021). Study reveals that periodontitis may cause oxidative damage in cardiac tissue, and crocin improves periodontitis-induced degenerative changes in heart tissue, which is associated with its antioxidant properties (Kocaman et al., 2021).

In addition, periodontitis and T2DM are characterized by increased mitochondrial oxidative stress production, which has been associated with a greater risk of cardiovascular diseases (Masi et al., 2019). Reduced ROS is associated with improved endothelial function and accompanied by better metabolic control in patients with T2DM and periodontitis (Masi et al., 2019). ROS could represent a novel therapeutic target to prevent cardiovascular disease in T2DM (Masi et al., 2019; Lee et al., 2020).

### 4.2 Periodontitis and diabetes mellitus

Diabetes mellitus is a metabolic disorder caused by an increased need for insulin. It is characterized by a relative or absolute under secretion of insulin, or insulin resistance, which results in decreased metabolism of carbohydrates, fat and protein, and higher than normal blood glucose levels in patients (Kurtalic et al., 2020; Sun et al., 2022). Oxidative stress is a common feature of both T1DM and T2DM, and elevated biomarkers of oxidative stress can be detected in blood, urine and tissues, including pancreas of patients with DM (Miki et al., 2018). T2DM is the most common subtype of diabetes, being present in 85%–90% of patients with a diagnosis of diabetes (Miki et al., 2018; Magiera et al., 2022). T2DM and periodontitis are two biologically linked diseases that often coexist in complex interaction (Luong et al., 2021). Most importantly, both diseases have similar mechanistic themes, such as chronic inflammation and oxidative stress (Luong et al., 2021). Alteration in the oral microbiome composition, which may activate the host inflammatory response and lead to irreversible oxidative stress, is a common finding in both diseases (Luong et al., 2021).

Studies on rats provided substantial evidence that both local and systemic oxidative damage and nuclear factor-E2-related factor 2 (Nrf2) downregulation are involved in the aggravation of periodontitis by DM (Li et al., 2018). Gene and protein expression of Nrf2 was significantly downregulated in diabetic periodontitis (Li et al., 2018). Compared to controls, periodontitis significantly increased local oxidative damage (increased expression of 3-nitrotyrosine, 4-hydroxy-2-nonenal, and 8-hydroxydeoxyguanosine). On the other hand, diabetes significantly increased systemic oxidative damage and suppressed antioxidant capacity (increased expression of MAD, decreased superoxide dismutase activity) (Li et al., 2018; Nishikawa et al., 2020). The concurrent development of periodontitis and diabetes was found to synergistically exacerbate local and systemic oxidative damage. This result correlates closely with greater periodontal destruction in diabetic periodontitis (Bogdan et al., 2020).

Some studies also suggest that increased systemic oxidative stress due to periodontitis activates systemic inflammatory signaling pathways that may influence the development of diabetes (Allen et al., 2011). Oral administration of curcumin and rutin, alone or in combination, can reduce oxidative stress and improve antioxidant status in rats with hyperglycemic periodontitis (Iova et al., 2021). Furthermore, MDA concentrations in blood and gum tissue have been shown to correlate with catalase activity (Iova et al., 2021).

### 4.3 Periodontitis and chronic kidney disease

Periodontitis and chronic kidney disease share many common risk factors, including obesity, smoking, and age (Li L. et al., 2021). There is growing evidence of a strong link between periodontitis and kidney disease. The oxidative stress induced by periodontitis can have a negative impact on the kidneys (Palathingal et al., 2022). It has been reported that induced periodontitis causes histomorphological changes in renal tissues, brush border disruption in the renal tubules, and changes associated with increased oxidative stress in the kidneys (França et al., 2017).

Antioxidants showed a protective effect against impaired liver and kidney function caused by experimental periodontitis (Li L. et al., 2021; Kose et al., 2021). In a mouse model of gingival sulcus, local induction of periodontitis with LPS and proteases increased hexanoyl-lysine (HEL) expression in the gingiva, leading to increased levels of HEL in serum and 8-OHdG in kidney tissue (Li L. et al., 2021). Compared with animals without periodontitis, the MDA content in the kidneys of the group with periodontitis was significantly increased and the glutathione concentration was significantly reduced (Li L. et al., 2021).

Another study showed that resveratrol therapy improves the local redox balance of the gingiva in periodontitis and reduces circulating oxidative stress (Li et al., 2023). Meanwhile, reduction of oxidative stress may alleviate renal damage (Jiang et al., 2020). Thus, periodontitis may increase the concentration of circulating oxidative stress, which in turn may cause kidney damage.

### 4.4 Periodontitis and liver injury

Growing evidence suggests that oxidative stress can cause lipid peroxidation, protein oxidation, DNA damage and mitochondrial dysfunction, and play a central role in liver injury (Huo et al., 2017). The antioxidant compound has been shown to decrease levels of damage marker enzymes such as aryl hydroxylase, gamma-glutamyl transferase, and adenosine deaminase in rat liver tissue, and ROS-induced lipid peroxidation in primary rat hepatocytes (Butnariu et al., 2022). These findings indicate that oxidative stress plays an important role in liver injury.

Recently, both animal and clinical studies have shown that periodontitis is associated with elevated levels of ROS in the blood, a condition that may be detrimental to liver health (Manjeu et al., 2022). According to previous study, the oxidative stress observed in periodontitis could induce a decrease in hepatic GSH, increasing oxidative imbalance and causing liver damage (Pessoa et al., 2018). The combination of ethanol and ligature-induced periodontitis was found to cause higher concentrations of HEL and 8-OHdG in the rat liver in comparison with ethanol exposure alone (Zieba et al., 2021). Supporting the notion, another rat model of periodontitis, the ligature-induced model, showed a decrease in glutathione in the liver antioxidant, and increase in circulating level of HEL, which suggests a possible link between periodontitis-generated oxidants and liver damage (Kumar et al., 2017).

## 5 Conclusion

In summary, periodontitis causes an imbalance between oxidants and antioxidants, triggering a mechanism of oxidative stress pathological damage that not only damages periodontal tissues but also affects the development of systemic diseases. The study of the relationship between periodontitis and systemic diseases is of great significance in the prevention and treatment of many systemic diseases. It is expected to provide new therapeutic approaches to raise awareness of oral hygiene and thus help to provide new treatment options to reduce the risk of periodontitis-related comorbidities.
